# The Omega‐6 Lipid pathway shift is associated with neutrophil influx and structural lung damage in early cystic fibrosis lung disease

**DOI:** 10.1002/cti2.70000

**Published:** 2024-09-16

**Authors:** Lisa JM Slimmen, Jelle Y Broos, Badies HAN Manaï, Silvia C Estevão, Martin Giera, Gijs Kooij, Wendy WJ Unger, Hettie M Janssens

**Affiliations:** ^1^ Division of Respiratory Medicine and Allergology, Department of Paediatrics Erasmus MC Sophia Children's Hospital, Erasmus University Medical Centre Rotterdam The Netherlands; ^2^ Laboratory of Paediatrics, Infection and Immunity Group, Department of Paediatrics Erasmus University Medical Centre Rotterdam The Netherlands; ^3^ Department of Molecular Cell Biology and Immunology, MS Centre Amsterdam Vrije Universiteit Amsterdam, Amsterdam Neuroscience, Amsterdam UMC Amsterdam The Netherlands; ^4^ Center for Proteomics and Metabolomics Leiden University Medical Centre Leiden The Netherlands

**Keywords:** alveolar macrophages, cystic fibrosis, inflammasome, lipid mediators, neutrophils, translational immunology

## Abstract

**Objectives:**

In cystic fibrosis (CF), an imbalanced lipid metabolism is associated with lung inflammation. Little is known about the role that specific lipid mediators (LMs) exert in CF lung inflammation, and whether their levels change during early disease progression. Therefore, we measured airway LM profiles of young CF patients, correlating these with disease‐associated parameters.

**Methods:**

Levels of omega (ω)‐3/6 PUFAs and their LM derivatives were determined in bronchoalveolar lavage fluid (BALF) of children with CF ages 1–5 using a targeted high‐performance liquid chromatography–tandem mass spectrometry approach. Hierarchical clustering analysis was performed on relative LM levels. Individual relative LM levels were correlated with neutrophilic inflammation (BALF %Neu) and structural lung damage (PRAGMA‐CF %Disease). Significant correlations were included in a backward multivariate linear regression model to identify the LMs that are best related to disease progression.

**Results:**

A total of 65 BALF samples were analysed for ω‐3/6 lipid content. LM profiles clustered into an arachidonic acid (AA)‐enriched and a linoleic acid (LA)‐enriched sample cluster. AA derivatives like 17‐OH‐DH‐HETE, 5‐HETE, 5,15‐diHETE, 15‐HETE, 15‐KETE, LTB_4_ and 6‐trans‐LTB_4_ positively correlated with BALF %Neu and/or PRAGMA %Dis. Contrastingly, 9‐HoTrE and the LA derivatives 9‐HoDE, 9(10)‐EpOME, 9(10)‐DiHOME, 13‐HoDE, 13‐oxoODE and 12(13)‐EpOME negatively correlated with BALF %Neu and/or PRAGMA %Dis. 6‐trans‐LTB_4_ was the strongest predictor for BALF %Neu. 5‐HETE and 15‐KETE contributed most to PRAGMA %Dis prediction.

**Conclusions:**

Our data provide more insight into the lung lipidome of infants with CF, and show that a shift from LA derivatives to AA derivatives in BALF associates with early CF lung disease progression.

## Graphical abstract

In early cystic fibrosis lung disease, a switch in the ω‐6 lipid pathway towards arachidonic acid derivatives associates with increased neutrophilic influx and structural lung damage, highlighting the need for timely intervention that mitigates lung inflammation.

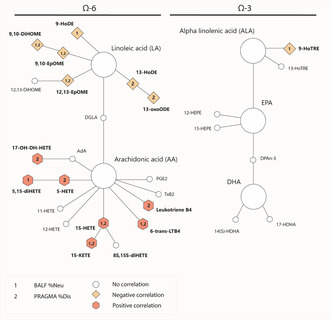

## Introduction

Cystic fibrosis (CF) is an autosomal recessive disease caused by mutations in the *CFTR* gene. The absence of functional CFTR protein leads to impaired transport of chloride and bicarbonate ions across the cell membrane, resulting in thick mucus excretions. Although CF is a multi‐organ disease, its most prominent feature is progressive lung damage caused by chronic airway infection and inflammation.^1^ Inflammation in CF lung disease is characterised by neutrophil influx into the airway lumen, which can already be detected in 3‐month‐old CF infants and is even observed in children with CF without respiratory symptoms.^2^, ^3^, ^4^, ^5^ The presence of neutrophils and their secreted enzymes, such as neutrophil elastase, associates with more rapid progression of structural lung damage in CF infants.^3^ In addition to increased inflammation, it is known that resolution of inflammation is impaired in CF lungs, further perpetuating chronic inflammation.^6^ Our recent findings suggest that the capacity of airway macrophages to counter inflammation and promote homeostasis is reduced during initiation of CF airway disease.^7^

Under normal conditions, cytokines, chemokines and bioactive lipid mediators (LMs) belonging to the ω‐3/6 pathways delicately orchestrate the different phases of an inflammatory response from onset towards its resolution, thereby enabling proper tissue recovery. The ω‐3/6 lipid pathways consist of polyunsaturated fatty acids (PUFAs) such as (among others) linoleic acid (LA), arachidonic acid (AA) and docosahexaenoic acid (DHA) and their LM derivatives. During the onset of inflammation, AA, an ω‐6 PUFA, is rapidly converted into a wide range of LMs such as prostaglandin E_2_ (PGE_2_) and leukotriene B_4_ (LTB_4_) that contribute to inflammation by increasing vascular permeability and promoting immune cell recruitment to the site of inflammation.^8^ This initial phase is followed by a process called LM class switching, characterised by the production of pro‐resolving LMs, such as lipoxin A_4_, that promote a return to homeostasis.^8^, ^9^

In CF, excessive activation of the AA pathway has been described before as reflected by increased AA:LA and/or AA(ω‐6):DHA(ω‐3) ratios compared to non‐CF controls, suggesting a (chronic) inflammatory state.^10^, ^11^ This ω‐6:ω‐3 imbalance has been demonstrated repeatedly in lung epithelial cell cultures and murine models, as well as plasma and sputum samples of CF subjects.^12^, ^13^, ^14^, ^15^, ^16^, ^17^, ^18^ However, it is unclear how accurately plasma and sputum reflect pathological processes in the airway lumen. As a result of the invasive nature of sampling, lipidomic analysis of PUFA‐derived LMs in bronchoalveolar lavage fluid (BALF) is scarce, yet higher levels of AA, as well as the AA‐derived 15‐KETE, are reported in adult CF BALF compared to non‐CF BALF.^19^, ^20^ We have previously reported abnormal levels of bioactive lipids in CF infants, although the relationship between early disease progression and changes in specific LMs remains to be further elucidated.^21^, ^22^

In this study, we therefore aim to further investigate the role of LMs in early CF lung disease. We measured LM profiles in BALF samples from CF infants (1–5 years), and correlated individual PUFA‐derived LMs to neutrophilic inflammation and structural lung damage. Investigating the potential relationship between the lung lipidome and CF lung disease will provide further insight into the pathogenesis of CF airway inflammation, and may provide new avenues for both monitoring and treating CF lung inflammation.

## Results

### Demographic data

A total of 65 study visits of 46 young CF patients from the I‐BALL cohort were included. Table 1 summarises the demographics per age group. The majority (96%) of samples were derived from subjects with at least one ΔF508 mutation. In general, *CFTR* mutations are divided into classes I–VI, of which IV‐VI result in partial CFTR protein function. These so‐called residual function mutations are typically associated with a milder disease phenotype.^1^ In our cohort, 22% of samples were from subjects with at least one known residual function mutation. Of note, eight samples were collected while the subject was receiving lumacaftor/ivacaftor treatment, with the treatment duration ranging from 7 days to 13.3 months. The incidence of BALF cultures positive for either bacterial or fungal pathogen increased with age, from 41% at age 1 to 78% at age 5 (*P* = 0.03, Table 1).

**Table 1:** Cohort demographics

	All (*n* = 65)	1 Year (*n* = 17)	3 Years (*n* = 22)	5 Years (*n* = 26)	*P*‐value^a^
Sex					
Male (%)	25 (38%)	5 (29%)	8 (36%)	12 (46%)	0.53
Female (%)	40 (62%)	12 (71%)	14 (64%)	14 (54%)	
*CFTR* mutation (%)					
Homozygous ΔF508	27 (41%)	6 (35%)	10 (45%)	11 (42%)	0.51
Heterozygous ΔF508	33 (51%)	8 (47%)	11 (50%)	14 (54%)	
No ΔF508	5 (8%)	3 (18%)	1 (5%)	1 (4%)	
Residual CFTR function (%)	16 (22%)	5 (29%)	4 (18%)	7 (26%)	0.68
Lumacaftor/ivacaftor therapy (%)	8 (12%)	0 (0%)	5 (23%)	3 (11%)	0.10
Duration^b^	6.2 (0.2–13.3)	—	6.7 (0.5–12.9)	5.6 (0.2–13.3)	
BALF culture					
Any pathogen	42 (65%)	7 (41%)	14 (64%)	21 (78%)	0.03
Bacteria^c^	41 (62%)	7 (41%)	13 (59%)	21 (78%)	0.03
*P. aeruginosa*	4 (6%)	0 (0%)	1 (5%)	3 (11%)	0.28
Fungal^d^	7 (12%)	1 (6%)	3 (14%)	3 (15%)	0.63
*Aspergillus* spp.	5 (8%)	1 (6%)	1 (5%)	3 (11%)	0.63

BALF, bronchoalveolar lavage fluid.

^a^ Age groups were compared using the Chi‐squared test.

^b^ Mean treatment duration (range) in months.

^c^ Cultures were positive for either one or several of the following pathogens: *Escherichia coli* (1), *Haemophilus influenzae* (18), *Moraxella catarrhalis* (5), *Pseudomonas aeruginosa* (4), *Staphylococcus aureus* (22) or other bacteria (6).

^d^ Cultures were positive for *Aspergillus fumigatus* (5) or *Penicillium* species.

### Neutrophilic inflammation and structural lung damage increase with age

**Figure 1:**
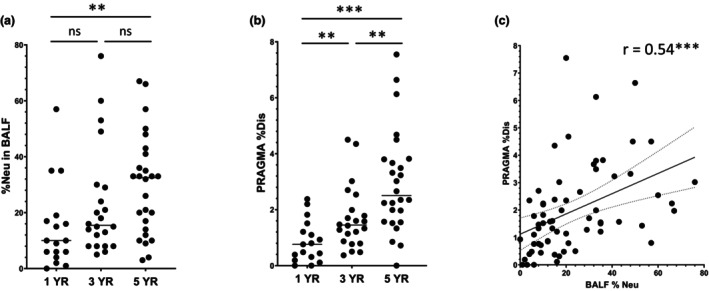
Progression of CF lung disease in early life. **(a)** BALF neutrophil count was obtained from clinical pathology reports and expressed as a percentage of total BALF leucocytes (BALF %Neu). **(b)** Structural lung damage was measured using the Perth–Rotterdam annotated grid morphometric analysis for CF (PRAGMA‐CF) score. PRAGMA‐CF %Disease (PRAGMA‐CF %Dis) is calculated as %bronchiectasis + %mucus plugging + %airway wall thickening. **(c)** Correlation between BALF %Neu and PRAGMA‐CF %Dis. Spearman correlation was used to assess the correlation of BALF %Neu and PRAGMA‐CF %Dis, and lines depict simple linear regression with a 95% confidence interval. The Mann–Whitney test was used to compare BALF %Neu and PRAGMA‐CF %Dis between age groups. Dots represent individual samples and horizontal lines depict median (ns, *P* > 0.05; **P < 0.005; ***P < 0.0001).

CF lung disease is characterised by persistent neutrophil influx to the airways, aggravating the inflammatory processes that ultimately lead to structural lung damage. The magnitude of this neutrophil influx is expressed as the percentage of neutrophils of total BALF leucocytes (BALF %Neu). Structural lung damage is summarised by the PRAGMA‐CF %Dis score measured on chest CT. In our cohort, both BALF %Neu (Figure 1a) and PRAGMA‐CF %Dis (Figure 1b) are significantly increased between ages 1 and 5 (*P* = 0.0035 & *P* < 0.0001, respectively). Moreover, BALF %Neu and PRAGMA‐CF %Dis show a strong correlation (*r* = 0.54, *P* < 0.001) (Figure 1c), similar to previous reports.^4^, ^23^ These data illustrate the progressive nature of CF lung disease, while also highlighting the high degree of variability in lung disease among CF infants.

### Hierarchical LM clustering shows two distinct patient populations

**Figure 2:**
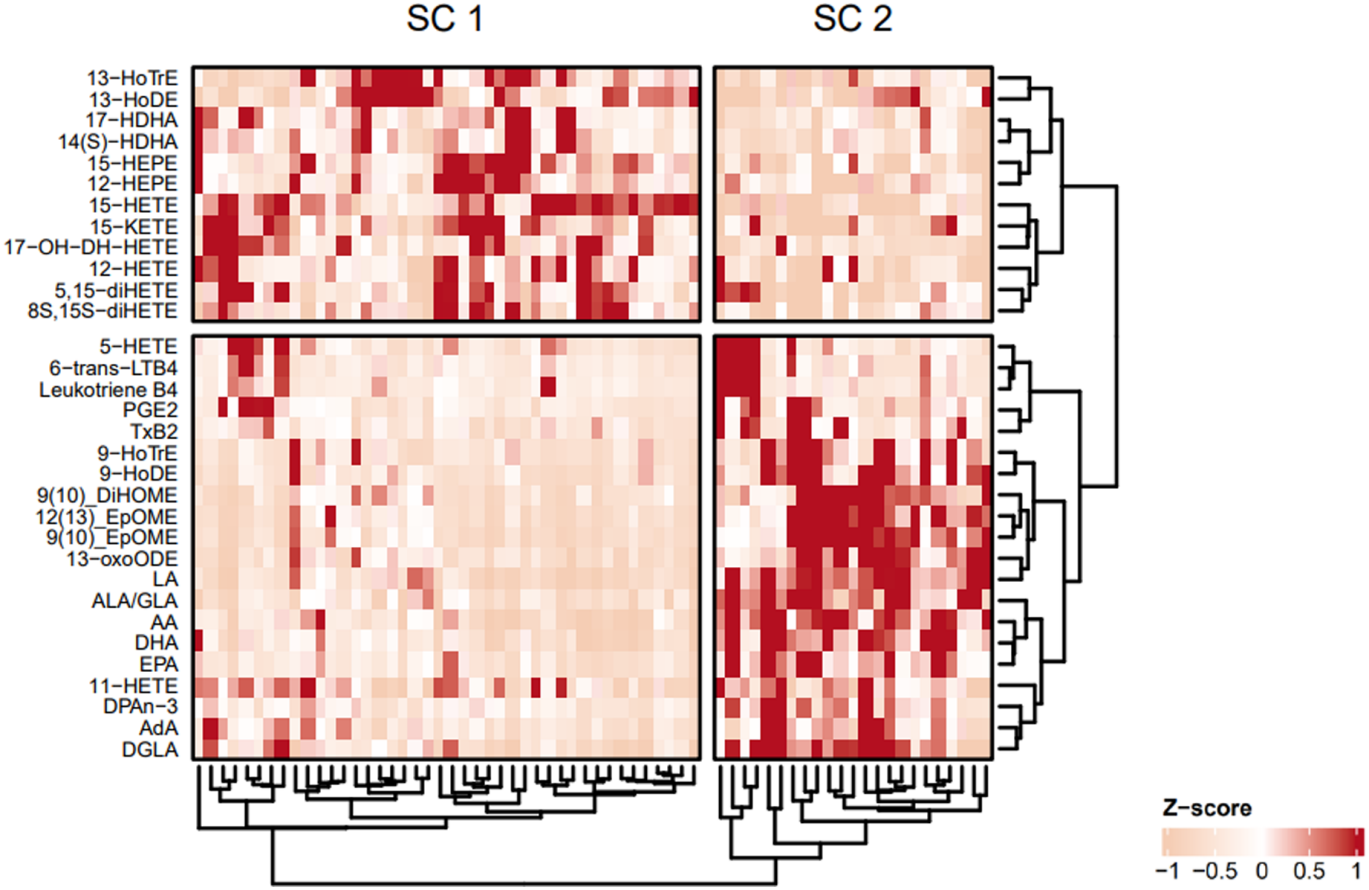
Two‐way hierarchical clustered using ω‐3/6 LM profiles show two distinct subject clusters. A heatmap with hierarchical cluster analyses (Euclidean distance) shows the distribution of CF subjects over two distinct sample clusters (SC1/2) associating with specific parts of the ω‐3/6 lipid pathways.

Elevated levels of AA and other ω‐6 LMs have been described in CF compared to non‐CF, and associate with dysregulated inflammatory processes.^10^, ^11^ However, the comparison between CF and non‐CF subjects accounts for neither the variability in lung disease among CF patients nor the changes that might occur in the early stages of lung disease progression. We therefore set out to investigate potential differences in ω‐3/6 LM profiles within our cohort. To account for the variability of lavage efficacy and subsequent BALF dilution, measured LM levels have been normalised using total area (TA) normalisation, resulting in relative lipid abundance rather than absolute concentrations. A two‐way hierarchical clustering method was used to visualise the cohort distribution of the measured LMs, generating two sample clusters (SC1 and SC2) and two lipid clusters (Figure 2). Samples were predominantly clustered based on the LM origin in the ω‐3/6 pathways (Supplementary figure 2). Subjects in SC1 displayed medium‐to‐high relative levels of AA and EPA/DHA derivatives (e.g. HETEs, HEPEs and HDHAs), and low relative levels of upstream ω‐3/6 PUFAs LA, AA, EPA and DHA and LA derivatives such as 9‐HoDE, 9(10)‐EpOME, 9(10)‐DiHOME and 12(13)‐EpOME. SC2 showed the exact opposite profile.

**Figure 3:**
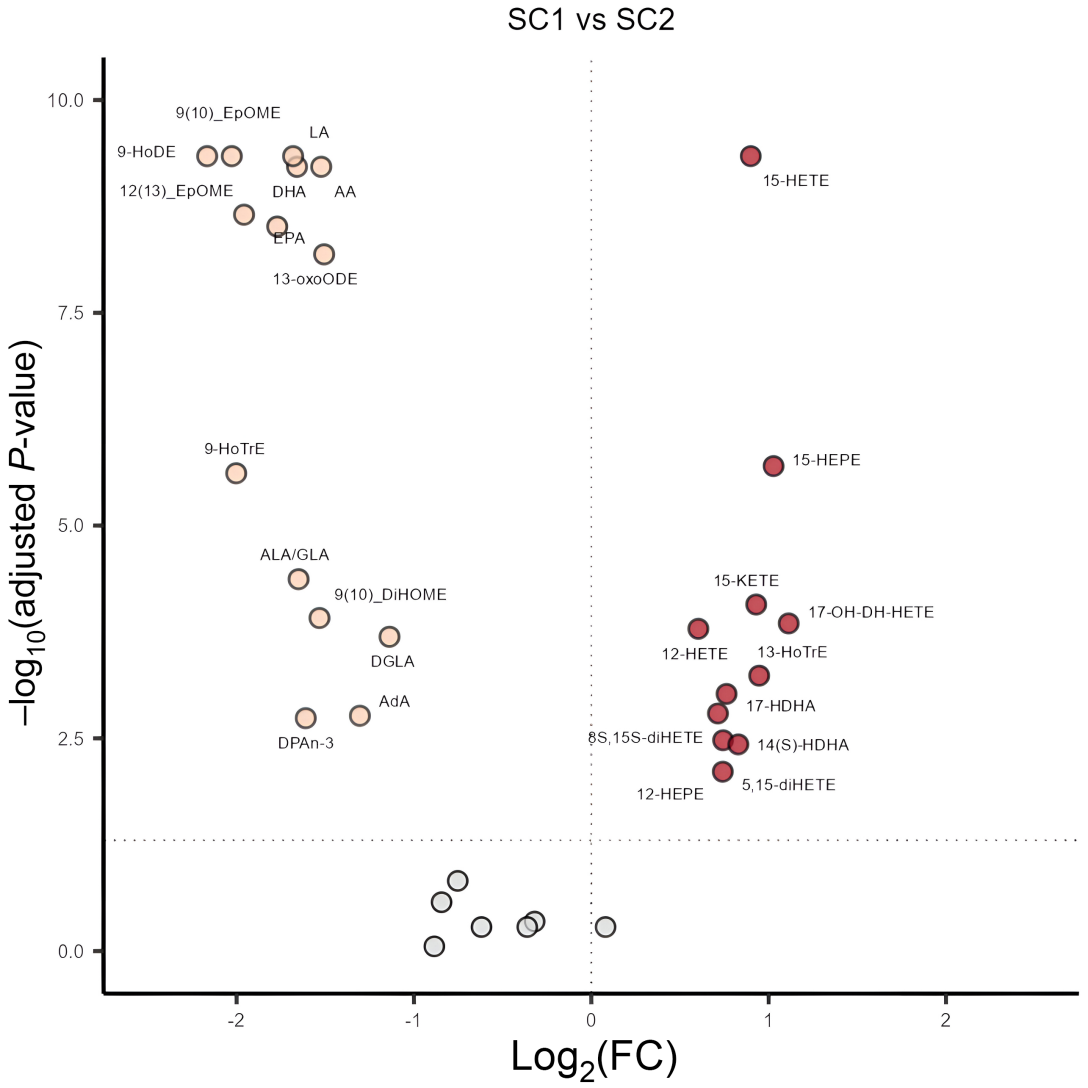
Comparison of relative LM levels between SC1 and SC2. A volcano plot showing the log2‐fold change in LMs between SC1 and SC2. Values are FDR corrected and dots represent individual LMs. Red colour indicates LMs whose levels are significantly higher in SC1 and yellow colour indicates LMs whose levels are significantly higher in SC2.

We next assessed the difference in relative LM levels and distribution of demographic and clinical variables between SC1 and SC2. Most relative LM levels were significantly different between SC1 and SC2, except for several AA derivatives such as PGE_2_ and LTB_4_ (Figure 3). Age, sex, positive microbiological cultures and residual CFTR function were evenly distributed between both clusters (Table 2). Intriguingly, all but one subject receiving lumacaftor/ivacaftor treatment were clustered into SC1, although this was not statistically significant. Lung disease parameters BALF %Neu and PRAGMA‐CF %Dis did not differ between SC1 and SC2 (Table 2).

**Table 2:** Comparison of demographic and clinical variables between sample clusters

	Cluster 1 (*n* = 42)	Cluster 2 (*n* = 23)	*P*‐value^a^
Age 1	11 (26%)	6 (26%)	0.89
Age 3	15 (36%)	7 (30%)	
Age 5	16 (38%)	10 (43%)	
Sex			
Male (%)	18 (43%)	7 (30%)	0.32
Female (%)	24 (57%)	16 (70%)	
Culture positive^b^	29 (69%)	13 (56%)	0.31
Residual function mutation	8 (19%)	8 (35%)	0.16
Lumacaftor/ivacaftor	7 (17%)	1 (0.04%)	0.24
BALF %Neu^c^, ^d^	20 (9.75–35.25)	13 (8–33)	0.12
PRAGMA‐CF %Dis^c^, ^e^	1.61 (0.67–3.02)	1.57 (0.87–2.35)	0.80

BALF, Bronchoalveolar lavage fluid; PRAGMA, Perth–Rotterdam annotated grid morphometric analysis.

^a^ Distribution of demographic characteristics between sample clusters was assessed using a Chi‐squared test (age, sex, culture result and residual function) or Fisher's exact test (lumacaftor/ivacaftor treatment).

^b^ BALF culture positive for any bacterial or fungal pathogen.

^c^ Median levels (IQR) and non‐parametric Mann–Whitney test.

^d^ Percentage neutrophils of total BALF leucocytes.

^e^ Total percentage of lung volume affected by bronchiectasis, mucus plugging and airway wall thickening.

### Correlations with neutrophilic inflammation and structural lung damage highlight an AA‐driven LM switch

**Figure 4:**
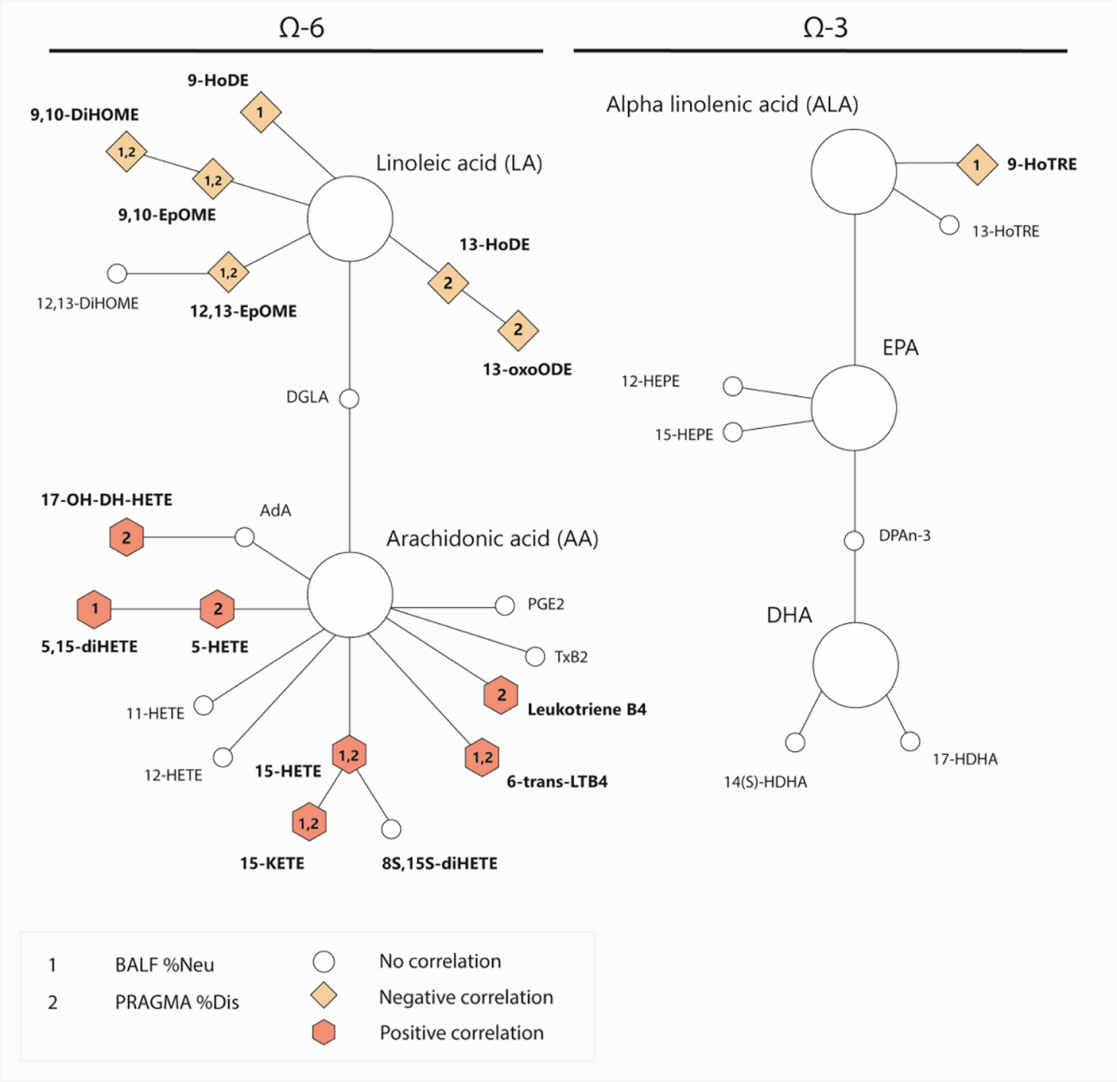
Visualisation of ω‐6 PUFA pathway correlations with BALF %Neu and/or PRAGMA‐CF %Dis. Red octagons show positive correlation and orange diamonds show negative correlations. Correlation with BALF %Neu, PRAGMA‐CF %Dis or both are indicated with the numbers 1 and/or 2, respectively.

Despite the lack of significant demographic changes between our two SC, individual LMs can still associate with disease parameters. As such, we next assessed the relationship between individual LMs and BALF %Neu and PRAGMA‐CF %Dis. Partial correlation plots were used in which gender, age, residual CFTR function, microbiological culture and lumacaftor/ivacaftor treatment were treated as confounding factors. Overall, positive correlations were found for AA‐derivatives 17‐OH‐DH‐HETE, 5‐HETE, 5,15‐diHETE, 15‐HETE, 15‐KETE, LTB_4_ and 6‐trans‐LTB_4_ with BALF %Neu, PRAGMA‐CF %Dis or both (Table 3, Figure 4). The opposite was the case for LA derivatives 9‐HoDE, 9(10)‐EpOME, 9(10)‐DiHOME, 13‐HoDE, 13‐oxoODE and 12(13)‐EpOME, and ALA derivative 9‐HoTrE, for which negative correlations with BALF %Neu and/or PRAGMA‐CF %Dis were observed (Table 3, Figure 4). These findings suggest that increased neutrophilic inflammation and structural lung damage coincide with a shift from LA derivatives towards AA derivatives.

**Table 3:** Correlation of lipid mediators with BALF %Neu and PRAGMA‐CF %Dis

Lipid mediator	Biosynthesis	%Neu	%Dis
**Ω‐6 pathway**			
Linoleic acid (LA)	—	−0.25	−0.19
Arachidonic acid (AA)	—	**−**0.21	0.03
AdA	—	−0.12	0.01
DGLA	—	−0.20	0.14
5‐HETE	ALOX‐5	0.09	**0.34** *
LTB_4_	ALOX‐5/LTA_4_H	0.15	**0.27** *
6‐trans‐LTB_4_	ALOX‐5/LTA_4_H	**0.36** **	**0.34** **
9‐HoDE	ALOX‐5/CYP/COX/FRO	**−0.31** *	−0.21
5,15‐diHETE	ALOX‐5/ALOX‐15	**0.27** *	0.23
12‐HETE	ALOX‐12	0.08	0.20
8,15‐diHETE	ALOX‐15	0.17	0.09
13‐HoDE	ALOX‐15	−0.14	**−0.33** *
13‐oxoODE	ALOX‐15 + dehydrogenase	−0.18	**−0.34** **
15‐HETE	ALOX‐15 + oxidation	**0.27** *	**0.29** *
15‐KETE	ALOX‐15	**0.38** **	**0.40** **
17‐OH‐DH‐HETE	ALOX‐15	0.20	**0.28** *
PGE_2_	COX‐1/2 + PGE_2_ synthase	0.02	0.16
TxB_2_	COX‐1/2 + TxAS	0.05	0.07
9(10)‐EpOME	CYP	**−0.33** *	**−0.30** *
9(10)‐DiHOME	CYP + sEH	**−0.32** *	**−0.26** *
12(13)‐EpOME	CYP	**−0.33** *	**−0.32** *
11‐HETE	CYP/FRO	0.13	0.18
**Ω‐3 Pathway**			
α‐Linoleic acid (ALA)	—	−0.13	0.08
EPA	—	−0.22	0.09
DHA	—	−0.23	0.02
DPAn‐3	—	−0.12	0.18
9‐HoTrE	ALOX‐5	**−0.28** *	0.01
12‐HEPE	ALOX‐12	0.03	0.05
14‐HDHA	ALOX‐12/ALOX‐15	0.00	0.15
13‐HoTrE	ALOX‐15	−0.15	−0.04
15‐HEPE	ALOX‐15	0.04	0.24
17‐HDHA	ALOX‐15	−0.02	0.13

Depicted values are Pearson *r* correlation coefficients. Bold values indicate a statistically significant correlation.

%Dis, total percentage of lung volume affected by bronchiectasis, mucus plugging and airway wall thickening; %Neu, Percentage neutrophils of total BALF leucocytes; ALOX, lipoxygenase; COX; cyclooxygenase; CYP, cytochrome P450; FRO, free radical oxygenation; LTA_4_H, LTA_4_ hydrolase; sEH, soluble epoxy hydrolase; TxAS; thromboxane A2 synthase. Bold values indicate *P*‐values < 0.05.

* *P* < 0.05.

** *P* < 0.005.

### 6‐trans‐LTB_4_ is a predictor of neutrophilic inflammation, while 5‐HETE and 15‐KETE are predictors of structural lung damage

To identify the LM(s) best related to the lung disease parameters of BALF %Neu and PRAGMA‐CF %Dis, a multivariate linear regression model was performed (Table 4). This model included all significant correlates of Table 3. Gender, age, residual CFTR function, microbiological culture and lumacaftor/ivacaftor treatment were included as covariates. Here, BALF %Neu was best predicted by age, residual CFTR function, microbiological culture and relative 6‐trans‐LTB_4_ levels. Relative 15‐KETE and 9‐HoTrE levels contributed to the BALF %Neu prediction model, although these were not significant. PRAGMA‐CF %Dis was best explained by age and gender, and relative levels of AA derivatives 5‐HETE and 15‐KETE. Positive microbiological culture, lumacaftor/ivacaftor treatment and relative 13‐oxoODE levels contributed to the model but did not meet the threshold for statistical significance. These findings reveal that lung disease progression in early CF, in addition to being correlated with demographic and clinical characteristics such as age, gender, severity of CFTR mutation and culture status, is also strongly associated with an increase in specific AA derivatives in the airways.

**Table 4:** Multivariate linear regression model for prediction of BALF %Neu and PRAGMA‐CF %Dis

Predictor	Adjusted *R* ^2^	*F*‐value	*P*‐value	Beta	*P‐*value
Predicting BALF %Neu					
15‐KETE	0.50	10.15	<0.001	0.19	0.06
9‐HoTrE				−0.18	0.06
6‐trans‐LTB4				0.20	**0.04**
Age (1 year)				−0.22	**0.02**
Residual CFTR function				−0.26	**0.008**
Culture positive^a^				0.38	**< 0.001**
Predicting PRAGMA‐CF %Dis					
5‐HETE	0.57	10.51	<0.001	0.32	**0.01**
15‐KETE				0.25	**0.009**
13‐oxoODE				−0.19	0.05
Age (1 years)				−0.57	**< 0.001**
Age (3 years)				−0.33	**0.001**
Gender (female)				0.26	**0.004**
Lumacaftor/ivacaftor treatment				−0.16	0.09
Culture positive^a^				0.17	0.06

BALF %Neu, Percentage neutrophils of total BALF leucocytes; PRAGMA‐CF %Dis, total percentage of lung volume affected by bronchiectasis, mucus plugging and airway wall thickening. Bold values indicate *P*‐values < 0.05.

^a^ BALF culture positive for any bacterial or fungal pathogen.

## Discussion

In a cohort of infants, we show that early CF lung disease progression, expressed as higher BALF %Neu and PRAGMA‐CF %Dis scores, coincides with a distinct shift in the ω‐6 lipid pathway, from LA derivatives to AA derivatives.

Imbalances in ω‐6 lipid metabolism, including an AA‐dominated environment, have been well described in CF and are thought to contribute to pulmonary symptoms.^10^, ^11^, ^16^ Much less is known, however, about the specific LMs involved, the differences between CF subjects and the LM changes that occur during lung early disease. As progressive lung disease is the most prominent cause of morbidity and mortality in CF, gaining more insight into the role of LMs in CF lung inflammation can contribute to new therapeutic avenues, as well as more precise disease monitoring tools.

While an increased AA:LA ratio has been described extensively in CF as compared to controls,^10^, ^11^, ^13^, ^14^ our findings reveal that not all children with CF display this increased AA:LA ratio to the same extent, suggesting that lipid imbalances typical for CF lung disease are at least in part acquired during the early disease stage, thus highlighting the need for timely intervention.

The finding that 6‐trans‐LTB_4_ was the strongest LM predictor for %BALF Neu is in line with the fact that LTB_4_ is a well‐described chemoattractant for neutrophils.^24^ We previously showed that LTB_4_ is higher in BALF of CF infants compared to non‐CF controls.^21^

Of all ω‐3/6 LMs that correlated with BALF %Neu and PRAGMA‐CF %Dis, we found that 15‐KETE was a predictor for PRAGMA‐CF %Dis, and was just shy of being a statistically significant predictor for BALF %Neu. 15‐KETE, also termed 15‐oxoETE, is an AA derivative that has been documented in BALF of adult CF patients before.^17^ Interestingly, in that study, 15‐KETE was nearly absent in BALF of non‐CF‐matched controls, suggesting a direct link with CF pathology. Several studies propose a beneficial role for 15‐KETE. In macrophage cultures, 15‐KETE promotes anti‐oxidant responses through an Nrf‐2‐dependent mechanism, inhibiting NF‐κB‐associated pro‐inflammatory responses.^25^ 15‐KETE was also found to prevent the apoptosis of pulmonary arterial smooth muscle cells through regulation of the Akt pathway in a rat model for pulmonary arterial hypertension.^26^ The overall increased abundance of HETEs, HEPEs and HDHAs in SC1 is indicative of increased 15‐lipoxygenase (ALOX15) activity, which is expressed by macrophages and is required for the production of pro‐resolving lipid mediators (also known as SPMs).^27^ We therefore speculate that the increased levels of 15‐KETE that we observe with advanced lung inflammation may reflect an anti‐inflammatory or pro‐resolving response that attempts to promote a return to homeostasis. Besides being a poor chemoattractant to monocytes,^28^ not much is known about the effect of 15‐KETE on tissue‐resident or infiltrating leucocytes. As many bioactive LMs can directly interact with receptors on immune cells, the possible immune‐modulatory effect of 15‐KETE warrants future investigation.

The lipidomic profile with more LA derivatives appeared to be negatively associated with both BALF %Neu and PRAGMA‐CF %Dis. LA‐derivatives like 9,10‐ and 12,13‐DiHOME, which are known to inhibit the neutrophil respiratory burst, could protect against structural lung damage.^29^ However, LA itself was found to promote neutrophilic respiratory burst,^30^ suggesting that an LA‐enriched lipidome might be beneficial not solely because of its suppression of neutrophilic inflammation, but a more general promotion of adequate neutrophilic function. Of note, therapeutic supplementation of LA has shown some positive effects on the growth of infant CF patients,^31^ but in a murine CF model actually led to higher AA levels and more neutrophil influx in BALF,^32^ suggesting that the interplay between PUFA metabolism and the progression of CF lung disease is complex and not easily mitigated by supplementation of LA. Another possible avenue of interest is the use of fenretinide, a synthetic retinoic acid derivative that is currently being explored as a therapeutic in CF. In CF mice, fenretinide decreased the AA:DHA ratio in plasma.^33^ However, the effects of fenretinide on the lung lipidome remain unknown. Its effects appear to be pleiotropic in nature and are still not fully understood.

Understanding of CF lung inflammation has evolved from merely the presence of chronic infection to a condition in which the lung's ability to counter inflammation and return to homeostasis is increasingly impaired.^6^ In this context, the role of pro‐resolving LMs in CF lung disease is of particular interest. Such LMs include several ω‐3/6‐derived LMs such as lipoxin A_4_, resolvins, protectins and maresins that promote resolution of inflammation in various ways.^8^, ^9^ Inability to induce an LM class switch towards these resolution‐promoting LMs may contribute to chronic inflammation. Unfortunately, as a result of their low abundance in *ex vivo* material, such mediators are difficult to measure in human samples, which was also the case in our dataset.

CFTR modulator therapy has improved clinical outcomes in CF,^34^, ^35^ but airway inflammation is still detectable under modulator treatment.^36^, ^37^ While our study was not set up to investigate the effect of lumacaftor/ivacaftor treatment on the lung lipidome, it is interesting to see that even with the low number of lumacaftor‐/ivacaftor‐treated subjects (8 of 65), this modulator appears to be a negative predictor, albeit not statistically significant, for PRAGMA %Dis. Still, with a mean BALF %Neu of 20.9% in lumacaftor‐/ivacaftor‐treated patients, which is well above the expected amount of neutrophils in a non‐inflamed lung,^38^ these findings confirm that airway inflammation persists despite lumacaftor/ivacaftor treatment. This is further highlighted by the fact that nearly all lumacaftor/ivacaftor‐treated subjects clustered into the AA‐enriched SC1. Together, these findings show lumacaftor/ivacaftor to have a disease‐stabilising, rather than a curative, effect on CF lung disease. Therefore, early initiation of more effective CFTR modulator treatment, such as elexacaftor/tezacaftor/ivacaftor, is highly anticipated. Additional studies investigating the effect of lumacaftor/ivacaftor treatment, as well as the more recently introduced elexacaftor/tezacaftor/ivacaftor treatment, on the LM profile and lung disease progression are needed.

The majority of LMs that were found to correlate with BALF %Neu and PRAGMA %Dis are biosynthesised by specific lipoxygenases such as ALOX‐5 or ALOX‐15, which would suggest increased activity of these enzymes. However, our data do not allow for direct conclusions on enzymatic activity, nor do they provide information on the cell types involved in the production of the LMs measured. To further complicate matters, many LMs can arise from non‐enzymatic reactions such as free radical oxidation. Therefore, further investigation into the chirality of some of these LMs would be needed in order to better discern the underlying regulatory mechanism of lipid production. CF is shown to lead to high levels of oxidative stress markers,^39^ which is to be expected with the neutrophilic inflammation involved.^23^ It is therefore reasonable to speculate that at least a portion of the LMs we measured is driven by reactive oxygen species.

Our study has several limitations. As a result of the cross‐sectional nature of our study, conclusions about causality cannot be made. Furthermore, while several subjects (*n* = 18) were included twice, our study is neither designed nor powered to draw any conclusions regarding longitudinal LM changes. Additionally, the use of relative abundance rather than absolute concentrations of LMs limits the conclusions that can be drawn from our data as an increased abundance of one LM will automatically result in a decreased abundance of another. The heterogeneity of BALF samples, and more specifically the variability in dilution of the samples, remains a challenge when comparing BALF components between subjects. Most parameters available for normalisation, such as protein content and leucocyte count, are themselves affected by inflammation and are therefore not suitable for normalisation in studies concerning inflammatory processes. However, as an inflammatory state is affected by the balance between pro‐inflammatory and anti‐inflammatory agents, the shift in relative abundance that we report is meaningful even with this limitation. Lastly, we do not have non‐CF controls to compare our data to. Unfortunately, the invasive nature of BALF collection does not allow for the collection of age‐matched healthy control material as a result of ethical constraints.

In summary, our data indicate that lung disease progression in early CF is associated with a ω‐3/6 PUFA shift from LA derivatives towards AA derivatives. Importantly, this shift is observed in clinically stable CF infants, highlighting the need for timely intervention with adequate treatment that is aimed at reducing airway inflammation.

## Methods

### Study design and subjects

Children diagnosed with CF aged 1–5 years were included in the early CF I‐BALL (inflammatory markers in bronchoalveolar lavage to predict early CF lung disease)monitoring programme at the Erasmus MC‐Sophia Children's Hospital in Rotterdam, the Netherlands. Children underwent bronchoscopy with bronchoalveolar lavage (BAL) and chest CT at ages 1, 3 and/or 5 while in stable clinical condition. Study samples were collected between May 2016 and November 2020. The study was approved by the institutional review board of the Erasmus MC and registered on clinicaltrial.gov (NCT02907788). Written informed consent was obtained from parents or legal guardians.

### Chest CT

A free‐breathing low‐dose chest CT was performed within 1 week of BALF collection. Structural lung disease was quantified using the standardised Perth–Rotterdam annotated grid morphometric analysis for CF (PRAGMA‐CF) scoring, performed by two independent observers.^40^ Diseased lung volume (PRAGMA %Dis), expressed as a percentage of total lung volume, was calculated as the sum of percentages with bronchiectasis, mucus plugging and airway wall thickening. Intra‐ and inter‐observer consistency scores for %Dis were 0.98 and 0.83, respectively.

### Bronchoalveolar lavage fluid collection and processing

Bronchoscopy was performed under general anaesthesia, and BALF was collected as described previously.^3^ Samples were placed on ice immediately after collection. BALF culture and cytology, including the percentage neutrophils of total BALF leucocytes (BALF %Neu), were performed as part of routine clinical care. Remaining BALF was homogenised with a 19G needle in 2.5 mm EDTA, centrifuged for 10 min at 800 *g* to pellet cells and then centrifuged for 10 min at 3000 *g* to obtain debris‐free supernatant that was used to measure LM content.

### Targeted lipid mediator analysis

BALF samples (400 μL) were quenched by adding 1.2 mL methanol (MeOH) and 4 μL internal standard solution consisting of LTB_4_‐d4, 15‐HETE‐d8, PGE_2_‐d4 and DHA‐d5 (50 ng mL^−1^) in MeOH. Samples were placed at −20°C for 20 min and centrifuged for 10 min at 16.200 *g* at 4°C after which LMs were extracted using solid‐phase extraction as described previously.^41^ Ω‐3/6 LM content of the samples was measured using a targeted HPLC‐MS/MS method.^41^ LMs were detected using their relative retention times (RRTs) together with characteristic mass transitions. These and other individually optimised parameters can be found in Supplementary table 1.

### Data handling and statistical analysis

LMs were excluded from the dataset if missing values were found in > 25% of the samples. Remaining missing values were imputed with 1/5th of the lowest value for each LM and data were normalised using total area (TA) normalisation to obtain relative values as previously described.^42^ In total, 32 LMs were used for statistical analyses which consisted of three parts, all of which were performed in Rstudio (version 4.2.2). Normality of the data was checked by visual inspection of histograms combined with Kolmogorov–Smirnov testing. A *P*‐value ≤ 0.05 was considered statistically significant.

First, a two‐way hierarchical cluster analysis (*ComplexHeatmap* package) was performed on all subject samples and data were clustered using a Euclidean distance method.^43^ Clinical characteristics of both SC were compared using Chi‐squared or Fisher's exact tests and relative LM levels were compared between SC using a non‐parametric Mann–Whitney *U*‐test. *P*‐values were corrected for multiple testing using an FDR correction.

Second, non‐normally distributed parameters (BALF %Neu and PRAGMA %Dis) were log transformed and all LMs were transformed with a Box–Cox transformation before partial correlation analyses. The relation between LMs and lung disease progression, expressed as BALF %Neu or PRAGMA %Dis, was assessed using Pearson correlations in which age, sex, residual CFTR function, culture positivity and lumacaftor/ivacaftor treatment were used as covariates.

Lastly, significant correlates of either BALF %Neu or PRAGMA %Dis were fed into respective multivariate linear regression models using a backward selection procedure with a removal *P*‐value of > 0.10. Age, sex, residual CFTR function, culture positivity and lumacaftor/ivacaftor treatment were included as covariates.

## Author contributions

**Lisa JM Slimmen:** Data curation; formal analysis; writing – original draft; writing – review and editing. **Jelle Y Broos:** Data curation; formal analysis; writing – original draft; writing – review and editing. **Badies HAN Manaï:** Data curation. **Silvia C Estevão:** Data curation. **Martin Giera:** Formal analysis; writing – review and editing. **Gijs Kooij:** Conceptualization; formal analysis; writing – review and editing. **Wendy WJ Unger:** Conceptualization; formal analysis; writing – review and editing. **Hettie M Janssens:** Conceptualization; formal analysis; writing – review and editing.

## Conflict of interest

The authors declare no conflict of interest.

## Supporting information


Supplementary table 1
Supplementary figures 1 and 2

## Data Availability

The data that support the findings of this study are available from the corresponding author upon reasonable request.
