# Supplementary material for: The Omega‐6 Lipid pathway shift is associated with neutrophil influx and structural lung damage in early cystic fibrosis lung disease
Source: Clin Transl Immunology. 2024 Sep 16;13(9):e70000. doi: 10.1002/cti2.70000 (PMC11403467; doi:10.1002/cti2.70000)
Supplement: Supplementary file 1 — Supplementary table 1 Supplementary figures 1 and 2 [file CTI2-13-e70000-s001.pdf]

Supplementary Table 1. List of lipid mediators detected using HPLC-MS/MS

Compound	Lipid group	LIPID MAPS ID	RT (min)	M/Z (Q1)	M/Z (Q3)	DP (V)	CE (V)	CXR (V)
9(S)-HoDE-d4	Internal standard (IS)	LMFA02000231	7.71	299.0	171.9	-85	-25	-18
DHA-d5	Internal standard (IS)	LMFA01030762	8.75	332	288.1	-75	-16	-13
PGE2-d4	Internal standard (IS)	LMFA03010008	4.9	355.1	193	-50	-25	-17
15-HETE-d8	Internal standard (IS)	LMFA03060080	7.8	327.2	226	-85	-18	-11
LTB4-d4	Internal standard (IS)	LMFA03020030	6.9	339.1	196.9	-70	-22	-19
LA	Ω-6 PUFA	LMFA01030120	8.8	279	261	-115	-28	-13
9(S)-HoDE	Ω-6 derivative	LMFA02000188	7.7	295	171	-130		
13(S)-HoDE	Ω-6 derivative	LMFA02000228	7.7	295	194.9	-110	-24	-21
13(S)-oxoODE	Ω-6 derivative	LMFA02000016	7.6	292	113	-105	-28	-13
9(10)-EpOME	Ω-6 derivative	LMFA02000037	7.99	294.9	123.1	-95	-20	-9
9(10)-DiHOME	Ω-6 derivative	LMFA02000229	7.14	312.9	170.9	-95	-29	-13
12(13)-EpOME	Ω-6 derivative	LMFA02000038	7.96	295	113	-110	-26	-13
DGLA	Ω-6 PUFA	LMFA010 30158	9.0	305.1	261.2	-85	-22	-13
AA	Ω-6 PUFA	LMFA010 30001	8.8	303	205.1	-155	-20	-11
AdA	Ω-6 derivative	LMFA010 30178	9.1	331.1	233	-130	-22	-11
17-OH-DH-HETE	hydroxy/hydroperoxyeicosatetraeinoic acid (HETE)	-	7.22	347.1	247	-110	-22	-27
5-HETE	hydroxy/hydroperoxyeicosatetraeinoic acid (HETE)	LMFA03060085	8	319.1	115	-65	-18	-11
5,15-DiHETE	hydroxy/hydroperoxyeicosatetraeinoic acid (HETE)	LMFA03060107	5.9	335	173.1	-55	-20	-11
11-HETE	hydroxy/hydroperoxyeicosatetraeinoic acid (HETE)	LMFA03060085	7.9	319.1	167	-70	-22	-15
12-HETE	hydroxy/hydroperoxyeicosatetraeinoic acid (HETE)	LMFA03060088	7.9	319.1	179	-65	-20	-23
15-HETE	hydroxy/hydroperoxyeicosatetraeinoic acid (HETE)	LMFA03060087	7.8	319.1	219.1	-55	-18	-9
15-KETE	hydroxy/hydroperoxyeicosatetraeinoic acid (HETE)	LMFA03060051	6.8	317	113	-10	-22	-5
8S,15S-DiHETE	hydroxy/hydroperoxyeicosatetraeinoic acid (HETE)	LMFA03060050	5.8	335	207.9	-55	-22	-17
Leukotriene B4	Leukotriene	LMFA03020001	6	335.1	195	-65	-22	-21
6-trans-LTB4	Leukotriene	LMFA03020013	5.8	335.1	194.9	-105	-22	-11
PGE2	Prostaglandine	LMFA03010003	3.8	351.2	271.1	-50	-22	-21
TxB2	Thromboxane	LMFA03030002	3.5	369.1	169	-55	-24	-15
ALA/GLA	Ω-3 PUFA	LMFA01030152	7.6	277	233	-90	-22	-29
9-HoTrE	Ω-3 derivative	LMFA02000024	7.35	292.9	170.9	-75		
13-HoTrE	Ω-3 derivative	LMFA02000051	7.4	292.9	195	-45	-24	-19
EPA	Ω-3 PUFA	LMFA01030759	8.55	301	202.9	-125	-18	
12-HEPE	hydroxy/hydroperoxyeicosapentaeinoic acid (HEPE)	LMFA03070031	7.6	317.0	179	-60	-18	-17
15-HEPE	hydroxy/hydroperoxyeicosapentaeinoic acid (HEPE)	LMFA03070032	7.5	317.1	219	-65	-18	-11
DPAn-3	Ω-3 PUFA	LMFA04000044	8.9	329.1	231.1	-50	-20	-17
DHA	Ω-3 PUFA	LMFA01030185	8.8	327.1	229.2	-115	-18	
14(S)-HDHA	hydroxy-docosahexaeinoic acid (HDHA)	LMFA04000058	7.95	343.1	204.9	-60	-18	-27
17-HDHA	hydroxy-docosahexaeinoic acid (HDHA)	LMFA04000012	6.9	343.1	245	-65	-16	-15

RT: retention time; m/z: mass-to-charge ratio; DP: declustering potential, CE: collision energy, CXP: collision exit potential

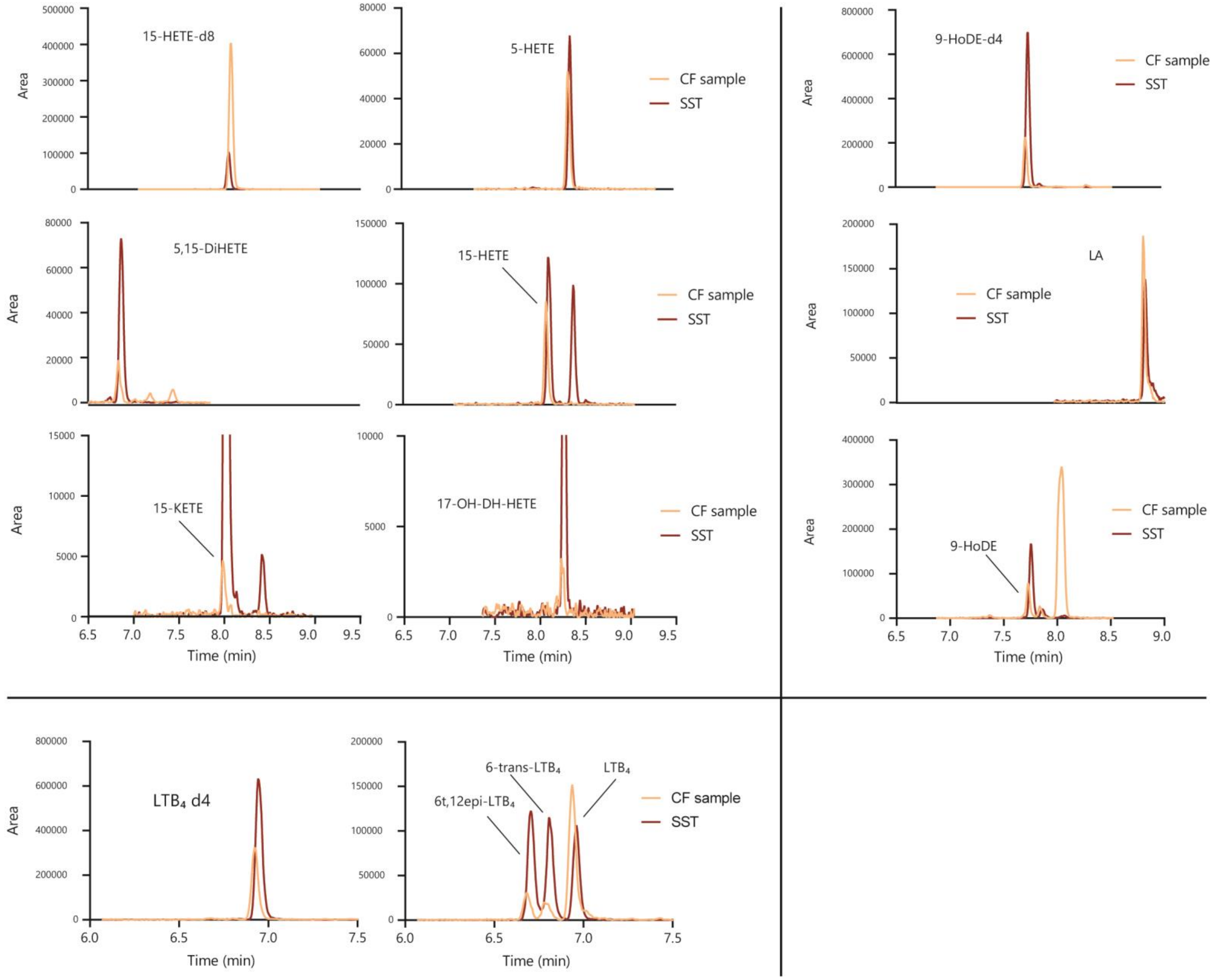

Supplementary figure 1. Representative chromatogram peaks of the most important lipids.
Each plot shows the chromatograms of one CF sample (orange) and the associated one system suitability test (SST) for each specific lipids. X-axis shows the retention time (RT) in minutes and the y-axis shows the measured area ratio. Lipids listed with the suffixes d3, d4 or d8 represent the used internal standards (IS).

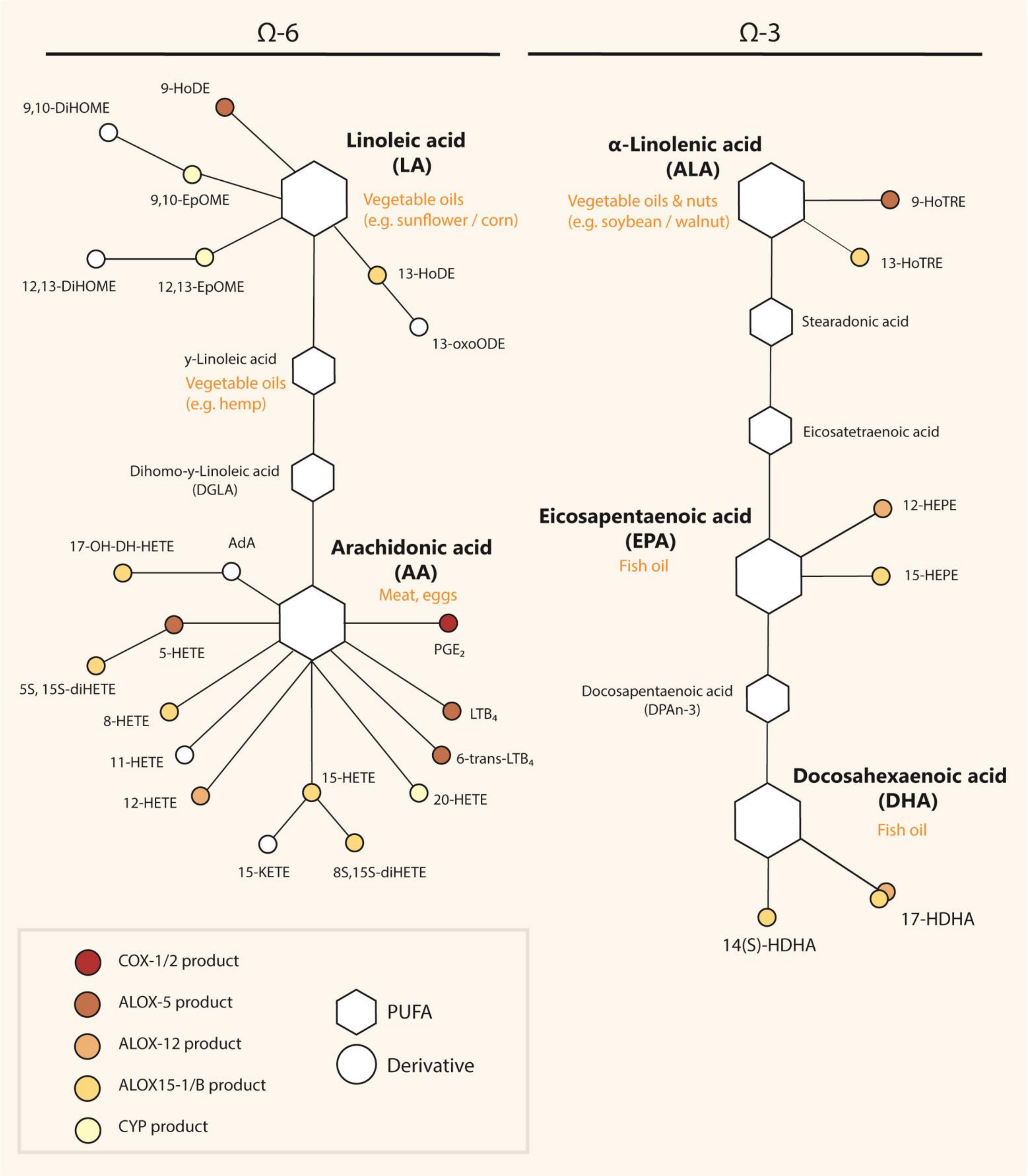

Supplementary Figure 2. Schematic overview of the Ω-3/6 lipid pathways
